# From Chronic Inflammation to Cancer: The Role of Trained Immunity in IBD-Associated Colorectal Carcinogenesis

**DOI:** 10.3390/medsci14020202

**Published:** 2026-04-17

**Authors:** Ferenc Sipos, Györgyi Műzes

**Affiliations:** Immunology Division, Department of Internal Medicine and Hematology, Semmelweis University, Szentkirályi Street 46, H-1088 Budapest, Hungary

**Keywords:** trained immunity, inflammatory bowel disease, colorectal cancer, chronic inflammation, epigenetic reprogramming, innate immune system, tumor microenvironment, metabolic reprogramming

## Abstract

Trained immunity is a concept that is currently in development and refers to the long-term functional reprogramming of innate immune cells in response to microbial or inflammatory stimuli. This process serves a dual purpose in the gastrointestinal tract, contributing to chronic inflammatory conditions like inflammatory bowel disease and maintaining host defense. The production of pro-inflammatory mediators is augmented by epigenetic and metabolic changes that are induced by the persistent activation of innate immune cells, which is triggered by microbial components and damage-associated signals. Although this increased responsiveness may initially be protective, sustained activation leads to tissue damage, epithelial barrier dysfunction, and chronic inflammation. These mechanisms are significant contributors to colorectal carcinogenesis, particularly in colitis-associated cancer. Through the activation of oncogenic signaling pathways, the establishment of a pro-tumorigenic microenvironment, and an increase in oxidative stress, trained immunity also influences tumor development. Additionally, the systemic reprogramming of hematopoietic progenitor cells has the potential to exacerbate inflammation and facilitate the progression of tumors. The identification of epigenetic and metabolic biomarkers associated with trained immunity can lead to novel diagnostic opportunities. Targeting metabolic and epigenetic pathways, as well as regulating the intestinal microbiota, is a promising therapeutic approach that could enhance the effectiveness of treatments for colorectal cancer while minimizing adverse effects on the immune system. Nevertheless, it is necessary to maintain a delicate equilibrium to suppress pathological inflammation without compromising protective immune responses. In general, trained immunity may represent a potentially relevant mechanistic link between chronic inflammation and colorectal cancer; however, its role remains context-dependent and not yet fully defined.

## 1. Introduction

Inflammatory bowel diseases (IBDs), which include Crohn’s disease (CD) and ulcerative colitis (UC), are chronic relapsing inflammatory disorders of the gastrointestinal tract. They are characterized by dysregulated immune responses to the intestinal microbiota in genetically predisposed individuals [1]. Long-term inflammation of the mucosa not only causes tissue damage over time, but it also greatly increases the risk of developing colorectal cancer (i.e., colitis-associated cancer; CAC) [2]. The mechanisms linking chronic intestinal inflammation and tumorigenesis are complex, involving interactions among epithelial cells, the microbiome, and both innate and adaptive immune responses [2]. Recently, the concept of trained immunity has emerged as a novel framework that may assist in elucidating the extent to which persistent innate immune activation contributes to the development of cancer and chronic inflammation in the intestine [3,4].

Chronic inflammation is a well-established driver of colorectal carcinogenesis and plays a central role in tumor initiation, promotion, and progression [2]. Persistent inflammatory signaling contributes to genomic instability, epigenetic alterations, and dysregulated cell proliferation, while also shaping the tumor microenvironment toward a pro-tumorigenic state. Inflammatory mediators such as cytokines, chemokines, and reactive oxygen species (ROS) promote DNA damage and support malignant transformation [2,3,4].

Importantly, trained immunity should be distinguished from chronic inflammation as a broader biological process. While trained immunity represents a specific form of innate immune reprogramming, many features of intestinal inflammation and tumorigenesis are driven by overlapping but not identical mechanisms [3,4,5,6,7]. Throughout this review, we aim to differentiate between evidence directly supporting trained immunity and findings derived from general inflammatory or tumor microenvironment (TME) studies.

In the past, immunological memory was considered a unique characteristic of adaptive immunity. Contrarily, there is an increasing body of evidence that innate immune cells can endure long-term functional reprogramming as a result of exposure to inflammatory stimuli or microbial components [5]. This phenomenon is termed “trained immunity.” Mihai G. Netea and colleagues systematically elucidated this concept, demonstrating that innate immune cells, including monocytes, macrophages, and natural killer (NK) cells, can exhibit heightened responsiveness upon secondary stimulation [5,6]. Trained immunity is induced by mechanisms such as metabolic and epigenetic reprogramming, resulting in enduring modifications to gene expression that exacerbate inflammatory responses, for instance, increased production of pro-inflammatory cytokines and enhanced phagocytic activity in innate immune cells [7].

The intestinal mucosa is continuously exposed to microbial antigens and inflammatory mediators in the context of IBD, which can create an environment that may significantly promote the induction of trained immunity [8,9]. The persistent activation of macrophages and other myeloid cells within the intestinal lamina propria can result from the repeated stimulation of innate immune cells by bacterial components, damage-associated molecular patterns (DAMPs), and cytokines [9]. These immune cells that have been trained demonstrate an increased production of pro-inflammatory cytokines, ROS, and chemokines, which may contribute to the chronic inflammatory state that is an identifying feature of IBD [4]. Additionally, there is a growing body of evidence that indicates that trained immunity can be achieved at the hematopoietic stem and progenitor cell level in the bone marrow. This phenomenon can result in sustained alterations in myelopoiesis and the continuous generation of hyperresponsive innate immune cells that migrate to the intestine [10].

Although trained immunity may improve host defense against pathogens and initially serve a protective function, its long-term consequences in chronic inflammatory diseases such as IBD may be detrimental [11]. Persistent innate immune activation can facilitate the accumulation of DNA damage within intestinal epithelial cells, increased oxidative stress, and epithelial barrier dysfunction [12,13,14]. These processes are widely acknowledged as the primary agents of inflammation-associated carcinogenesis [15]. The production of growth factors, angiogenic mediators, and matrix-remodeling enzymes is also induced by chronic inflammatory signaling, which creates a microenvironment that is conducive to the initiation and progression of tumors [16]. Therefore, the correlation between colorectal cancer (CRC) and IBD offers a compelling context in which trained immunity may affect disease outcomes. The secretion of cytokines such as tumor necrosis factor (TNF)-α, interleukin (IL)-6, and IL-1β by hyperactivated macrophages and other innate immune cells can promote tumorigenesis [17]. These cytokines activate oncogenic signaling pathways in epithelial cells and support the survival and proliferation of tumor cells [16,18]. Furthermore, the formation of a tumor-promoting inflammatory niche within the intestinal microenvironment may be facilitated by trained myeloid cells, which can enhance the secretion of pro-inflammatory cytokines and create a supportive environment for tumor growth [19,20]. However, it is crucial to emphasize that the contribution of trained immunity to colorectal carcinogenesis remains incompletely understood, and current evidence is often indirect or context-dependent. In this review, we distinguish between evidence directly derived from IBD-associated colorectal carcinogenesis, findings extrapolated from studies on sporadic colorectal cancer or general inflammatory mechanisms, and more speculative, hypothesis-generating concepts. This distinction is critical for accurately interpreting the current state of the field.

Future research will focus on conducting a detailed investigation of the role of trained immunity in chronic inflammation-induced colorectal carcinogenesis. Understanding this process could lead to the development of new treatments for IBD, thereby reducing the development of CAC. It may also facilitate the reduction in detrimental inflammation and regulate the immune system’s memory, thereby augmenting the efficacy of current medications and improving the management of chronic inflammatory disorders. Ultimately, these discoveries could improve patients’ quality of life and health status.

## 2. Literature Search Strategy

This narrative review is the result of a structured, non-systematic literature search that was conducted in the PubMed, Scopus, and Web of Science databases. The following keywords were employed to screen publications up to March 2026: “trained immunity”, “innate immune memory”, “colorectal cancer”, “colitis-associated cancer”, “inflammatory bowel disease”, “ulcerative colitis”, “Crohn’s disease”, “tumor-associated macrophages”, “tumor microenvironment”, “epigenetic reprogramming,” and “immunometabolism”.

Articles that were published exclusively in English were incorporated. Review papers and original research articles were both taken into account. Recent, high-quality studies and those that offered mechanistic or translational insights into trained immunity were prioritized. In the event that direct evidence was scarce, pertinent studies from related disciplines (e.g., macrophage biology or chronic inflammation) were incorporated and interpreted with caution. The selection of studies was determined by their conceptual contribution and relevance to the subject matter, rather than by formal systematic inclusion criteria.

## 3. Trained Immunity and Its Role in Intestinal Physiology

Traditionally, the immune system has been divided into two primary components: innate and adaptive immunity. Germline-encoded pattern recognition receptors (PRRs) are used by the cells of the innate immune system to identify pathogens and tissue injury [21,22]. These receptors detect a variety of molecular patterns associated with pathogens and damage (i.e., PAMPs and DAMPs). Adaptive immunity was long regarded as the major mechanism responsible for immunological memory, while innate immunity was thought of as a rapid but non-specific defense system without memory. This difference was due to the presence of DCs and antigen-specific T and B lymphocytes [23]. Nevertheless, this paradigm has substantially changed in the past decade with the recognition of “trained immunity [5,6,7].” The concept is a mechanism in which innate immune cells undergo a long-lasting functional reprogramming as a result of exposure to PAMPs and DAMPs. Innate immune mechanisms can be activated by specific infections and vaccinations (e.g., bacillus Calmette–Guérin (BCG) vaccine) to provide comprehensive protection against other pathogens [5,6]. In contrast, the phenomenon known as “LPS tolerance” is also an adaptation of cellular responses to an external stimulus that can be induced by modest doses of lipopolysaccharide (LPS) and other Toll-like receptor ligands [24]. However, it results in a decrease in the inflammatory response to a subsequent stimulation, which can lead to an increased susceptibility to infections due to the body’s reduced ability to mount an effective immune response.

### 3.1. Metabolic and Epigenetic Reprogramming as the Basis of Trained Immunity

Trained immunity is fundamentally driven by the long-term metabolic and epigenetic reprogramming of innate immune cells [7,25,26,27,28]. These interrelated mechanisms reshape cellular function and establish a memory-like state that facilitates improved responsiveness upon secondary stimulation.

Epigenetic reprogramming constitutes a central mechanism of trained immunity [29,30,31]. It involves stable modifications in chromatin structure, including histone methylation and acetylation, which regulate gene accessibility without altering the DNA sequence. Essential epigenetic modifications, such as histone H3 lysine 4 trimethylation (H3K4me3) and histone H3 lysine 27 acetylation (H3K27ac), accumulate at promoters and enhancers of genes that encode pro-inflammatory cytokines and antimicrobial molecules [29,30,31]. These alterations establish a permissive chromatin configuration that allows for rapid and amplified transcriptional responses upon reactivation.

In parallel, metabolic reprogramming is essential for the induction and maintenance of trained immunity. Upon activation, innate immune cells alter their metabolism to enhance glycolysis, even under aerobic conditions, akin to the Warburg effect observed in cancer cells [32,33,34,35]. This metabolic transition provides not only rapid ATP generation but also critical biosynthetic intermediates that promote cell activation. Furthermore, processes like glutaminolysis and cholesterol synthesis produce metabolites (e.g., fumarate and acetyl-CoA) that directly affect epigenetic enzymes and chromatin remodeling [32,33,34,35].

Metabolic and epigenetic processes are tightly interconnected. Metabolites generated during cellular metabolic rewiring function as cofactors or regulators of chromatin-modifying enzymes, so connecting cellular metabolism to transcriptional regulation. This integration ensures the stability and persistence of trained immune responses over time.

Collectively, metabolic and epigenetic reprogramming constitute the mechanistic foundation of trained immunity, enabling innate immune cells to acquire long-lasting functional adaptations in response to environmental stimuli.

While the fundamental mechanisms of trained immunity are defined by these interconnected metabolic and epigenetic processes, their functional consequences extend to multiple innate immune cell populations and are supported by extensive experimental evidence.

These molecular mechanisms translate into the functional reprogramming of multiple immunocompetent cells, including monocytes, macrophages, dendritic cells (DCs), natural killer (NK) cells, innate lymphoid cells (ILCs), and tissue-resident stem cells [7]. These functional modifications enable innate immune cells to respond more swiftly and robustly to a diverse array of pathogens or inflammatory signals.

Despite monocytes’ short lifespans, trained immunity can last three to twelve months. This persistence is attributed to the reprogramming of bone marrow hematopoietic stem and progenitor cells, which generate progeny with enhanced inflammatory potential. In some cases, heterologous protection following live vaccinations may persist for up to five years [25]. Notably, recent studies suggest that trained immunity may even exert transgenerational effects [26,27].

Many endogenous signals and microbial components can induce trained immunity. The BCG vaccine and β-glucans derived from fungal cell walls are among the best-characterized stimuli. These signals are recognized by pattern recognition receptors (PRRs), including Toll-like receptors (TLRs) and C-type lectin receptors (CLRs) [7,28], triggering intracellular signaling cascades that ultimately drive the metabolic and epigenetic reprogramming processes described above.

At the molecular level, trained immunity is closely associated with chromatin remodeling [29,30,31]. Histone methylation and acetylation regulate gene accessibility, with key activating marks such as H3K4me3 and H3K27ac accumulating at promoters and enhancers of genes encoding inflammatory cytokines, chemokines, and antimicrobial molecules. This epigenetic priming enables rapid transcriptional responses upon secondary stimulation [29,30,31].

In parallel, metabolic reprogramming plays a critical role in shaping trained immunity [32,33,34,35]. Activated innate immune cells exhibit enhanced glycolysis even under aerobic conditions, similar to the Warburg effect observed in cancer cells. This metabolic shift supports rapid ATP production and provides biosynthetic intermediates necessary for cellular activation. Additionally, pathways such as glutaminolysis and cholesterol synthesis generate metabolites that influence chromatin structure and the activity of epigenetic enzymes [32,33,34,35].

These tightly interconnected metabolic and epigenetic alterations collectively underpin the establishment and maintenance of trained immunity (Table 1).

### 3.2. Trained Immunity in Intestinal Homeostasis

Trained immunity is crucial to intestinal physiology, one of the most complex immunological environments in the body. Microbial antigens from commensal microbiota and pathogens bombard the gut mucosa. Immune tolerance and defense must be balanced to maintain intestinal homeostasis [36]. The lamina propria comprises gut macrophages, DCs, and ILCs that sense microbial signals and coordinate immune responses. Intestinal innate immune cells are capable of developing immunity from microbial ligands in this setting. Commensal bacteria produce LPSs, peptidoglycans, and short-chain fatty acids that interact with host PRRs [37,38]. These signals can promote long-term gut immune response modifications by affecting mucosal immune cell activity. Thus, trained immunity may be a physiological mechanism by which the host adapts to its microbiota, responding faster and more effectively to microbial assaults.

The physiological function of intestinal trained immunity partly relies on intestinal macrophages, characterized by high phagocytic activity but minimal inflammatory cytokine production [39]. This feature enables them to tolerate commensal microorganisms. Environmental inputs in the gut can affect macrophage function and generate trained-like states that increase tolerance [40]. Gut macrophages may immediately respond to infections while preserving tissue homeostasis under steady-state settings due to this adaptability [41].

Intestinal epithelium derived cytokines, antimicrobial peptides, and metabolic signals also regulate immune cell activity and train immunity [42]. Epithelial factors, including the IL-1 family of cytokines and growth factors, can activate mucosal macrophages and DCs [9,43]. Epigenetic changes that modify epithelial cell reactivity to microbial stimuli may also show innate immune memory, potentially enhancing the ability of these cells to respond more effectively to subsequent microbial encounters [44,45].

Intestinal microbiota is crucial for trained immunity and providing extra regulation. Microbial metabolites, including butyrate, propionate, and acetate, alter immune cell metabolism and epigenetic control, affecting trained immunological responses [46,47]. Dysbiosis, or microbial changes, can affect gut innate immune programming. Normal microbiota disruption increases inflammation and impairs mucosal barrier function [48,49].

In conclusion, trained immunity is a crucial innate immune system adaptation that allows long-term functional reprogramming in response to environmental signals. Immune, epithelial, and microbial cells form a dynamic network in the intestine to maintain mucosal homeostasis and prevent infections. The processes influencing the gut’s trained immunity may reveal intestinal physiology and new treatments for inflammatory and neoplastic intestinal disorders.

## 4. The Pathogenic Aspects of Trained Immunity in Inflammatory Bowel Disease: Diagnostic and Therapeutic Perspectives

In the context of IBD, there is increasing experimental and clinical evidence supporting a role for trained immunity in sustaining chronic intestinal inflammation.

In the intestinal mucosa, innate immune cells are perpetually exposed to microbial ligands that are derived from the commensal microbiota. In healthy individuals, mucosal macrophages and DCs retain the ability to respond to pathogens while maintaining a state of controlled responsiveness that promotes tolerance to commensal organisms. However, the dysregulation of trained immunity can cause intestinal inflammation. Repeated stimulation by microbial components such as LPS, peptidoglycan fragments, and fungal β-glucans may induce trained immunity in monocytes and macrophages [4,11]. Prolonged activation of trained innate immune cells can lead to chronic inflammatory disorders like IBD [4]. Pro-inflammatory cytokines and ROS can damage the intestinal epithelium, promote immune cell infiltration, and perpetuate inflammation and tissue injury [4,11].

At the molecular level, epigenetic modifications that increase the transcriptional accessibility of pro-inflammatory genes are the defining characteristics of trained immunity in IBD. The rapid and amplified production of inflammatory mediators is facilitated by histone modifications, including increased H3K4me3 and H3K27ac at the promoters of cytokine genes [4,50,51]. As a result, trained macrophages generate elevated concentrations of pro-inflammatory cytokines (i.e., TNF-α, IL-1β, and IL-6), which are primary contributors to intestinal inflammation [52]. Within the intestinal microenvironment, these cytokines contribute to the disruption of the epithelial barrier, the recruitment of additional immune cells, and the amplification of inflammatory signaling pathways [53,54,55].

The pro-inflammatory phenotype is further maintained by metabolic reprogramming. The energy and biosynthetic intermediates required for prolonged activation are provided by innate immune cells that undergo trained immunity, which transition toward glycolysis and increased glutamine metabolism [56,57]. This metabolic shift in the chronically inflamed intestinal mucosa contributes to tissue injury and reinforces the persistence of inflammatory responses [58].

It is crucial to note that trained immunity may not be restricted to circulating monocytes; it may also involve hematopoietic stem and progenitor cells in the bone marrow [59], a phenomenon often termed “central trained immunity” [60]. These progenitor cells may be reprogrammed by chronic inflammatory signals that originate from the gut, resulting in the sustained production of hyperresponsive myeloid cells that migrate to the intestinal mucosa [4,61]. It is possible that the relapsing nature of IBD and the persistence of inflammation even after transient clinical remission can be attributed to this systemic component of trained immunity.

Comprehending the role of trained immunity in inflammatory bowel disease offers innovative diagnostic possibilities. Although several features of trained immunity, including epigenetic and metabolic reprogramming, have been proposed as potential biomarkers, it is important to note that most of these candidates remain at a preclinical or early translational stage. Robust clinical validation in human cohorts is currently lacking. Certain histone modification patterns in circulating monocytes or altered metabolic profiles may indicate the degree of innate immune activation in patients with IBD [52,62,63,64]. Potential biomarkers, such as cleaved H3T22, H3K4me3, Rme2sym, phosphorylated H3S10, and H3K9/K14ac, could improve the precision of clinical and laboratory metrics and therefore help the assessment of disease progression and therapeutic efficacy [63,64,65].

Furthermore, the identification of patient subgroups with unique inflammatory phenotypes may be facilitated by profiling trained immunity-related pathways. Using transcriptomic, epigenetic, and metabolic methods, it is possible to profile these pathways. Epigenetic profiling methods, such as ATAC-seq or ChIP-seq for histone modifications, can identify chromatin changes associated with innate immune memory, while transcriptomic analyses, such as RNA sequencing, can reveal inflammatory gene expression signatures. Furthermore, metabolic rewiring -such as altered TCA cycle activity or enhanced glycolysis- that characterizes trained immunity phenotypes can be detected through metabolomic analyses and cellular metabolic assays. By determining individuals who are more likely to benefit from therapies that target innate immune activation, this could facilitate a more tailored approach to treatment.

An emerging strategy in the management of IBD is the targeting of trained immunity from a therapeutic perspective. The majority of current treatments concentrate on the suppression of adaptive immune responses or the neutralization of critical inflammatory cytokines [66]. For example, biologic therapies that target TNF-α have become a fundamental component of treatment for both UC and CD. Nevertheless, the necessity of alternative therapeutic approaches is underscored by the fact that a significant number of patients either fail to respond or experience a decline in responsiveness over time [67].

The therapeutic targeting of trained immunity represents an emerging and conceptually attractive strategy; however, most approaches discussed below are supported primarily by preclinical or early translational evidence, and only limited data are available from clinical studies.

Pharmacological therapies targeting trained immunity-related pathways are being considered as potential treatments for macrophage-driven intestinal inflammation. Modulating the AMP-activated protein kinase (AMPK)–mechanistic target of rapamycin (mTOR) signaling axis, which is crucial to activated macrophage metabolic reprogramming, is a viable method [68,69,70]. In experimental colitis models, the AMPK activator metformin inhibits mTOR signaling, reduces proinflammatory cytokine production, and improves intestinal barrier integrity [71]. Rapamycin, an mTOR inhibitor, has shown promise in preclinical and limited clinical investigations of refractory CD, pointing toward the fact that targeting metabolic signaling pathways may reduce chronic intestinal inflammation [72,73].

NOD-like receptor family pyrin domain containing 3 (NLRP3) inflammasome activation is a key inflammatory mechanism in IBD pathogenesis, promoting IL-1β and IL-18 maturation and release. IBD patients’ intestinal tissues have excessive NLRP3 activation, and inhibiting this pathway reduces intestinal inflammation in animal models [71,74].

Although Z-VAD-fmk (Z-Val-Ala-Asp(OMe)-fluoromethylketone; a pan-caspase inhibitor) is mostly employed in experiments, pharmacological targeting of the inflammasome pathway has potential for medicinal development [75].

Cholesterol production pathways are associated with learned innate immune responses [76]. In particular, the mevalonate pathway reprograms macrophage epigenetics and metabolism [77]. Statins like fluvastatin suppress inflammatory cytokine production and may modulate the immune system by inhibiting this route [78]. Statins may reduce disease activity and hospitalization rates in IBD patients, but more clinical research is needed [79].

Additional experiments try to disrupt metabolic alterations that activate trained macrophages. By preventing the metabolic switch needed for trained immunity, glycolysis inhibitors like 2-deoxy-D-glucose or 3PO can reduce macrophage inflammatory responses [4,80]. However, its therapeutic applicability in humans remains at an early investigative stage.

Another promising strategy involves focusing on the upstream triggers of trained immunity, particularly microbial signals originating from the intestinal microbiota [4]. Dietary interventions, probiotics, or microbiota-based therapies, which are intended to restore microbial homeostasis, may indirectly affect innate immune programming and reduce chronic inflammatory activation.

**Figure 1:**
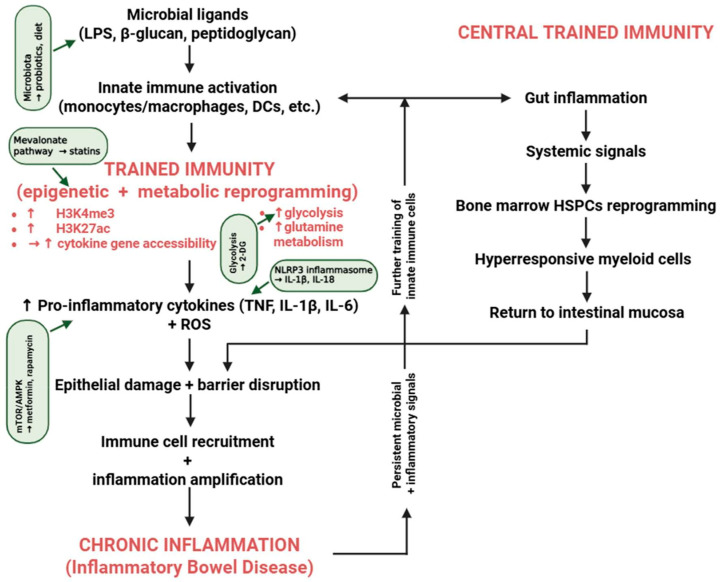
Mechanisms for and therapeutic targeting of trained immunity in intestinal inflammation. In the intestinal mucosa, trained immunity is induced by the repeated exposure of innate immune cells to microbial ligands. This immune phenomenon is characterized by epigenetic (e.g., increased H3K4me3 and H3K27ac) and metabolic reprogramming (e.g., enhanced glycolysis and glutamine metabolism). This results in the increased production of pro-inflammatory cytokines (TNF-α, IL-1β, and IL-6) and reactive oxygen species, which in turn contribute to the disruption of the epithelial barrier, the recruitment of immune cells, and the persistence of intestinal inflammation. Inflammatory bowel disease (IBD) is characterized by a self-perpetuating feedback loop that reinforces chronic inflammatory responses. At the same time, systemic signals have the ability to reprogram bone marrow progenitors, thereby fostering the continuous production of hyperresponsive myeloid cells (central trained immunity). The mevalonate pathway, metabolic reprogramming, the NLRP3 inflammasome, the AMPK–mTOR axis, and microbiota modulation are all potential therapeutic targets. The interactions shown in this figure represent a combination of evidence-supported mechanisms, proposed pathways, and speculative or hypothesis-generating concepts. While some processes are supported by experimental data, others are inferred from related fields, including inflammation biology and tumor microenvironment studies, and should be interpreted with caution. Figure was partly created with https://biorender.com/ (accessed on 19 March 2026).

Finally, the therapeutic modulation of trained immunity must be approached cautiously, as the complete suppression of innate immune memory could compromise host defense against infections. Consequently, it is probable that future therapies will endeavor to selectively reduce pathologically trained immune responses while maintaining protective immune functions. Mechanisms and therapeutic targets of trained immunity in intestinal inflammation are depicted in Figure 1.

## 5. The Pathogenic Role of Trained Immunity in Colorectal Cancer: Implications for Colitis-Associated and Sporadic Tumorigenesis

While the title of this review emphasizes IBD-associated colorectal carcinogenesis, this section extends the discussion to sporadic colorectal cancer. Notably, much of the evidence presented here is extrapolated from studies on TAMs, chronic inflammation, and the TME, and only limited data directly address trained immunity in human colorectal cancer.

Patients with chronic IBD have an elevated risk for the development of CAC [81,82]. In case of chronic intestinal inflammation, immunocompetent cells are continuously exposed to PAMPs, cytokines, and DAMPs. These stimuli have the potential to induce long-lasting epigenetic reprogramming in local or circulating innate immune cells. Trained macrophages and monocytes produce increased levels of pro-inflammatory cytokines, such as IL-1β, IL-6, and TNF-α. These cytokines have been demonstrated to promote epithelial proliferation, inhibit apoptosis, and activate oncogenic signaling pathways like STAT3 and NF-κB [19,52,83]. In colonic epithelial cells, genomic instability is exacerbated by the persistent production of inflammatory mediators. DNA damage and mutagenesis can be induced by ROS and RNS that are produced by activated macrophages [84]. Over time, the dysplasia-carcinoma transition is promoted by the accumulation of genetic alterations in intestinal epithelial cells within a pro-inflammatory milieu [85]. Consequently, trained immunity has been proposed as a contributing mechanistic link; however, direct evidence remains limited, and its relative importance compared to other inflammation-driven processes is still under investigation.

Trained immunity may play a role not only in CAC but also in the development of sporadic colorectal cancers, although this assumption is largely based on extrapolation from inflammation-driven and macrophage-centered studies. Even in individuals without IBD, the intestinal mucosa is constantly exposed to microbial and dietary stimuli that can reprogram innate immunity [4,9,86]. Intestinal dysbiosis is a common phenomenon in CRC patients [87]. The resulting persistent stimuli can activate trained immunity [4,10]. Certain microorganisms produce metabolites or virulence factors that, through the activation of PRRs, can lead to the development of sustained inflammatory signaling in the TME [88,89].

Within colorectal tumors, trained immune cells, especially tumor-associated macrophages (TAMs), may influence multiple aspects of cancer progression. TAMs are particularly important in this context. These cells often display phenotypes that support tumor growth, angiogenesis, and immune suppression [90]. While TAMs are central players in the TME, their functional states should not be equated directly with trained immunity. It is essential to understand that trained immunity does not merely duplicate traditional M1 polarization. Instead, it causes a unique metabolic and epigenetic reprogramming that leads to macrophage states that have some of the same properties as the canonical M1/M2 phenotypes, but not all of them [91]. This reprogramming may result in contradictory pro- and anti-neoplastic activities within the TME, whereas metabolic imprinting enhances the functional diversity of tumor-associated macrophages. It should be noted that the following observations are primarily derived from studies on TAMs and the TME, and do not directly demonstrate trained immunity in the context of CRC.

Trained immunity influences the function of TAMs in CRC through coordinated epigenetic and metabolic reprogramming. The persistent accumulation of H3K4me3 at the promoters of inflammatory genes, such as CXCL9 and CXCL10, is a critical characteristic that is typically induced by β-glucan [92]. This modification establishes a long-lasting epigenetic memory that increases the expression of pro-inflammatory genes upon restimulation. Metabolic restructuring is essential for the preservation of this condition. The trained phenotype is stabilized by the accumulation of fumarate, which inhibits KDM5 histone demethylases and prevents the elimination of H3K4me3 marks as a result of increased aerobic glycolysis and glutaminolysis, but its therapeutic applicability in humans, particularly in colorectal cancer, remains at an early investigative stage [32,34]. Simultaneously, the elevated levels of H3K27ac at enhancers and promoters facilitate an open chromatin configuration, which in turn enhances transcriptional responsiveness [93]. Further regulatory layers encompass the targeting of epigenetic complexes, such as WDR5/MLL1, to specific loci by lncRNA to promote H3K4me3 deposition, as well as the regulation of DNA methylation dynamics by DNMT and TET enzymes [94,95]. For instance, the maintenance of a pro-tumor M2-like phenotype can be impaired by Tet2 deficiency [96,97]. The sustained functional activity of trained macrophages is facilitated by enhanced creatine metabolism, although it is not directly epigenetic [98]. Based on the above, it is evident that epigenetic programming associated with trained immunity may enhance the ability of macrophages to produce cytokines and growth factors that promote the survival and invasion of tumor cells [99]. However, the use of epigenetic modulators in this context is currently exploratory and not yet supported by clinical evidence.

Additionally, trained myeloid cells may contribute to the recruitment and expansion of immunosuppressive populations such as myeloid-derived suppressor cells, thereby limiting effective anti-tumor immune responses [10].

In addition to local reprogramming within the TME, trained immunity may function at the bone marrow level, referred to as “central trained immunity.” In this situation, hematopoietic stem and progenitor cells endure prolonged epigenetic and metabolic remodeling due to persistent inflammatory signals from the gut [60]. This systemic imprinting leads to the ongoing production of hyperresponsive myeloid cells, which maintain a functionally active TME [3]. As a result, TAMs are not only affected by local signals but also by a preconditioned myeloid compartment [3,60]. This pathway may contribute to persistent inflammation that fosters tumor development, diminishes therapy efficacy, and leads to disease recurrence. The explanation of this phenomenon is that freshly recruited myeloid cells maintain a trained, pro-inflammatory, or immunomodulatory phenotype.

These observations are consistent with the idea that trained immunity could contribute to sporadic colorectal carcinogenesis; however, this remains largely hypothetical and requires further direct experimental validation.

Understanding the pathogenic function of trained immunity in CRC can lead to novel diagnostic applications. Epigenetic fingerprints linked to trained immune cells may function as indicators for cancer risk or disease progression; however, current evidence is largely derived from in vitro and animal studies, and their clinical applicability remains to be established. For example, particular histone modification patterns or transcriptional profiles in circulating monocytes (e.g., H3K4me1, H3K4me3, H3K27ac, or PFKFB3-a glycolysis activator) may indicate systemic innate immune activation linked to tumor progression [100,101]. Likewise, metabolic changes linked with trained immunity -like heightened glycolysis or modified lipid metabolism- might be observable in peripheral blood cells and could act as markers of early tumor-related inflammation, but their specificity and reproducibility in human disease contexts remain unclear [102,103]. Biomarkers linked with trained immunity may assist in differentiating between inflammation-driven and sporadic CRC or in identifying people at an elevated risk for colitis-associated malignancy [104,105]. This information could be particularly beneficial for monitoring individuals with long-term IBD, as it could aid in the early detection of neoplastic transition.

Overall, while biomarker development in this field is conceptually promising, it remains in an early phase and requires systematic clinical validation before implementation in patient care.

Therapeutically, the targeting of trained immunity is a promising but still emergent strategy in the management of CRC. Modulation of metabolic pathways that maintain trained immune activation is one potential approach. The inflammatory potential of trained macrophages within the TME could potentially be reduced by inhibitors of glycolysis (e.g., 2-deoxy-D-glucose), glutamine metabolism (e.g., the prodrug JHU083), or cholesterol synthesis (i.e., statins) [106,107,108,109,110].

In the same vein, drugs that alter epigenetic enzymes responsible for histone acetylation (e.g., epigallocatechin-3-gallate) or methylation (e.g., inhibition of the histone methyltransferase G9a) may reverse the persistent transcriptional activation that is a hallmark of trained immunity [10,111].

An additional approach entails the targeting of the upstream triggers of innate immune reprogramming. The microbial signals that induce chronic trained immune activation may be reduced by modifying the intestinal microbiota through dietary interventions, probiotics, or microbiome-based therapies; however, their role in modulating trained immune responses in colorectal carcinogenesis remains largely hypothetical [112]. Patients diagnosed with CRC linked to inflammation may discover these approaches especially relevant.

It is intriguing that trained immunity can also be utilized therapeutically to improve anti-tumor immunity. Certain immunomodulatory compounds or vaccines that are capable of inducing beneficial trained immune responses may encourage innate immune cells to more effectively recognize and eliminate tumor cells [19]. The difficulty is in the selective promotion of protective anti-tumor responses while preventing chronic inflammatory processes that promote tumor progression.

In conclusion, trained immunity represents a potentially relevant, but still incompletely defined, mechanism linking microbial exposure, chronic inflammation, and colorectal carcinogenesis. However, this relationship remains largely inferential and requires further mechanistic validation, particularly in human systems. A key limitation of the field is the relative scarcity of direct human evidence linking trained immunity to colorectal carcinogenesis, with most current insights derived from in vitro systems or extrapolated from related inflammatory contexts. These aspects are discussed in more detail in the Controversies and Open Questions section.

**Figure 2:**
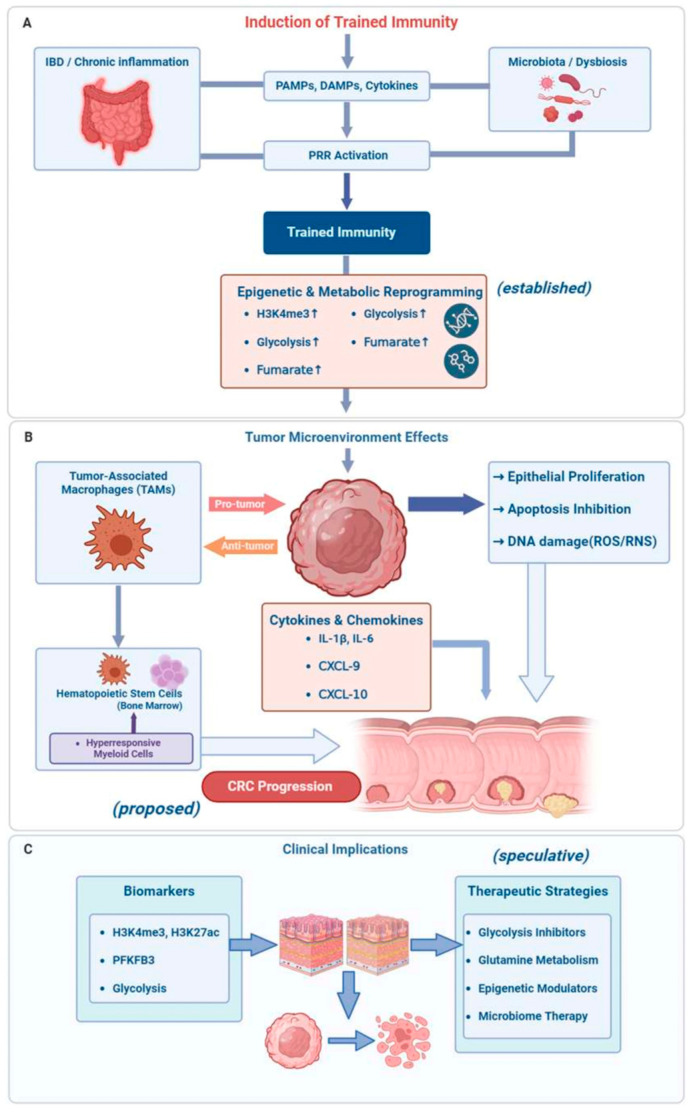
Proposed and extrapolated mechanisms linking trained immunity to colorectal cancer. (**A**) Through continuous exposure to pathogen-associated molecular patterns (PAMPs), damage-associated molecular patterns (DAMPs), and cytokines, chronic intestinal inflammation in inflammatory bowel disease (IBD) and persistent microbial and dietary stimuli in non-IBD individuals induce trained immunity. These signals activate pattern recognition receptors (PRRs) on innate immune cells, resulting in a long-lasting epigenetic and metabolic reprogramming that is characterized by an increase in H3K4me3 and H3K27ac histone modifications, enhanced glycolysis and glutaminolysis, and the accumulation of metabolites such as fumarate. (**B**) Trained macrophages, such as tumor-associated macrophages (TAMs), produce an increased amount of pro-inflammatory cytokines (e.g., IL-1β, IL-6, TNF-α) and chemokines (e.g., CXCL9, CXCL10), which contribute to a microenvironment that promotes tumor growth. Ultimately, these mediators elicit DNA damage through reactive oxygen and nitrogen species (ROS/RNS), activate oncogenic pathways such as STAT3 and NF-κB, and stimulate epithelial proliferation, resulting in genomic instability and the progression of colorectal cancer (CRC). Simultaneously, the continuous generation of hyperresponsive myeloid cells that further influence the TME is sustained by central trained immunity at the level of hematopoietic stem and progenitor cells in the bone marrow. In addition, trained immunity induces immunosuppressive mechanisms, such as the recruitment of myeloid-derived suppressor cells. (**C**) In the clinical setting, trained immunity-associated epigenetic and metabolic signatures may function as biomarkers for the risk and progression of cancer. Therapeutic strategies that target trained immunity include microbiome-based interventions, epigenetic regulators, and modulation of cellular metabolism (e.g., glycolysis and glutamine metabolism inhibitors). In contrast, the induction of trained immunity under controlled conditions may improve antitumor responses. The interactions shown in this figure represent a combination of evidence-supported mechanisms, proposed pathways, and speculative or hypothesis-generating concepts. While some processes are supported by experimental data, others are inferred from related fields, including inflammation biology and tumor microenvironment studies, and should be interpreted with caution. Figure was partly created with https://biorender.com/ (accessed on 20 March 2026).

The long-term reprogramming of innate immune cells, which alters the TME and promotes disease progression, may affect both CAC and sporadic CRCs. Innovative therapeutic strategies aimed at modulating innate immune memory in CRC, as well as novel biomarkers for cancer risk assessment, may result from ongoing research into the molecular mechanisms and clinical implications of trained immunity. Proposed mechanisms and therapeutic targets of trained immunity in colorectal carcinogenesis are summarized in Figure 2.

## 6. Controversies and Open Questions

Despite the growing interest in trained immunity in IBD, CAC, and CRC, several important conceptual and mechanistic questions remain unresolved. A major limitation of the current field is the imbalance between experimental and human evidence. Much of the available data derives from in vitro systems or animal models, whereas direct evidence in human CAC/CRC remains deficient. Consequently, the extent to which findings from experimental systems translate to human disease is uncertain. This restraint is further compounded by the frequent extrapolation of findings from IBD and general inflammatory biology to sporadic CRC, where direct evidence for trained immunity remains limited.

Another key unresolved issue is whether trained immunity predominantly exerts pro- or anti-tumor effects [17,98]. While enhanced cytokine production and antigen presentation may facilitate anti-tumor responses, sustained activation of trained myeloid cells may also perpetuate chronic inflammation, promote angiogenesis, and reinforce immunosuppressive networks, highlighting the strong context dependency of these processes [113]. Similarly, the role of classical inducers such as β-glucan remains ambiguous. Although β-glucan-driven training has demonstrated anti-tumor efficacy in experimental settings, its effects in CRC—particularly in the context of chronic inflammation—may be variable or even detrimental [114].

The durability and reversibility of trained immune phenotypes also remain incompletely understood. While trained immunity is often described as long-lasting, it is unclear to what extent these phenotypes are reversible, particularly within the TME. This question is closely related to the plasticity of TAMs, as it remains uncertain whether these cells can be effectively reprogrammed therapeutically or whether they become functionally fixed over time [115].

An additional layer of complexity arises from the relative contribution of tissue-resident versus bone marrow-derived immune programming. In addition to local microenvironmental signals within the gut, systemic reprogramming of hematopoietic progenitors may contribute to the continuous supply of preconditioned myeloid cells, potentially sustaining tumor-associated inflammation and influencing disease persistence or recurrence [116]. However, the interplay between these local and systemic mechanisms remains poorly defined.

A fundamental conceptual challenge is the distinction between trained immunity and general inflammatory activation. Many features attributed to trained immunity, including enhanced cytokine production and metabolic reprogramming, are also characteristic of conventional inflammatory responses. Clear experimental criteria to distinguish these processes in complex in vivo settings, particularly in human disease, are still lacking.

Finally, it remains unclear whether trained immunity represents a distinct biological program in CRC or whether it largely overlaps with established frameworks of TAM polarization. Although certain epigenetic and metabolic features may be shared, TAM phenotypes are shaped by diverse tumor-derived signals. Determining whether trained immunity provides additional explanatory value beyond conventional TAM classification remains an important open question.

Collectively, these uncertainties highlight that, despite its conceptual appeal, trained immunity in colorectal carcinogenesis remains an emerging and not yet fully defined framework. Addressing these questions will be essential for clarifying its biological significance and for translating this concept into clinically relevant diagnostic and therapeutic strategies.

## 7. Future Perspectives

Trained immunity has transformed our comprehension of the innate immune system by revealing that innate immune cells can undergo enduring functional reprogramming, which plays a role in chronic inflammation and malignancy. In the gastrointestinal tract, this process is crucial for maintaining mucosal immune activation in inflammatory bowel disorders, where ongoing exposure to microbial and inflammatory stimuli leads to sustained activation of macrophages and monocytes. This persistent inflammatory condition harms epithelial cells and increases the likelihood of tumor development, potentially resulting in colorectal cancer. Additionally, trained immunity transcends local tissues to the bone marrow, where it reprograms hematopoietic progenitors, leading to the sustained generation of hyperresponsive myeloid cells that maintain intestinal inflammation.

Future study in this field will probably concentrate on many critical areas. First, additional elucidation of the molecular processes governing trained immunity in the gastrointestinal tract is required. Progress in epigenomics, single-cell transcriptomics, and metabolomics will provide a more comprehensive examination of the reprogramming of innate immune cells in response to microbial and inflammatory stimuli. These technologies may assist in identifying certain epigenetic markers, transcriptional networks, and metabolic pathways that facilitate trained immune responses in intestinal disorders.

Second, finding biomarkers linked to trained immunity could help with risk assessment and disease surveillance. Epigenetic markers, metabolic profiles, or transcriptional patterns in circulating innate immune cells may function as biomarkers of chronic inflammatory activity or early neoplastic transformation. These indicators could be particularly valuable in monitoring patients with long-term IBD, as they are at a higher risk of developing CAC.

Third, the therapeutic modulation of trained immunity is a new and promising approach. Current treatments for inflammatory bowel disease mostly focus on adaptive immune responses or specific inflammatory cytokines. Nonetheless, therapies that modify innate immune programming may offer supplementary or alternative therapeutic strategies. Targeting metabolic pathways that maintain trained immune activation, altering epigenetic regulators, or manipulating the gut microbiome could potentially diminish pathological inflammation and restrict tumor-promoting immune responses.

It is also vital to remember that trained immunity helps to protect the host. Consequently, treatment interventions must achieve an optimal equilibrium between mitigating harmful chronic inflammation and preserving essential immune functions. Selective control of trained immune pathways, as opposed to global immune suppression, is likely to be the most effective strategy.

In summary, trained immunity serves as an essential link between environmental exposures, microbial signals, and immune control in the gastrointestinal tract. Dysregulation contributes to the pathogenesis of IBD and may facilitate CRC proliferation by perpetuating inflammatory signals and altering the TME. Continued research into the mechanisms and clinical implications of trained immunity will deepen our understanding of intestinal physiology and pathology, potentially leading to innovative diagnostic tools and targeted therapies aimed at improving outcomes for patients with chronic intestinal inflammation and colorectal cancer.

## Figures and Tables

**Table 1:** Metabolic and Epigenetic Mechanisms Underlying the Development of Trained Immunity [7,28,29,30,31,32,33,34,35]. The table summarizes key mechanisms associated with trained immunity and indicates whether the supporting evidence is derived from gut-specific studies or extrapolated from the broader trained immunity literature. The level of evidence varies across mechanisms and should be interpreted accordingly.

Mechanism	Description	Disease Relevance	Representative Mediators/Examples
Epigenetic reprogramming	Histone modifications and chromatin remodeling	IBD/CAC (direct evidence); CRC (extrapolated)	H3K4me3, H3K27ac
Metabolic reprogramming	Shift toward glycolysis and altered metabolism	CRC (extrapolated)	mTOR, HIF-1α, HK2
Cytokine production	Enhanced pro-inflammatory signaling	IBD (direct evidence); CRC (extrapolated)	IL-1β, TNF-α, IL-6
Myelopoiesis	Expansion of myeloid progenitors	Systemic inflammation (extrapolated)	GM-CSF, IL-1β
Tumor microenvironment interaction	Crosstalk with stromal and immune cells	CRC (extrapolated)	TAMs, TGF-β

## Data Availability

No new data were created or analyzed in this study. Data sharing is not applicable to this article.

## Abbreviations

The following abbreviations are used in this manuscript:

AMPK	AMP-activated protein kinase
ATP	Adenosine triphosphate
ATAC-seq	Assay for Transposase-Accessible Chromatin using sequencing
BCG	Bacillus Calmette–Guérin
CAC	Colitis-associated cancer
CD	Crohn’s disease
ChIP-seq	Chromatin immunoprecipitation sequencing
CLRs	C-type lectin receptors
CRC	Colorectal cancer
CXCL9	C-X-C motif chemokine ligand 9
CXCL10	C-X-C motif chemokine ligand 10
DAMPs	Damage-associated molecular patterns
DCs	Dendritic cells
DNMT	DNA methyltransferase
H3K27ac	Histone H3 lysine 27 acetylation
H3K4me1	Histone H3 lysine 4 monomethylation
H3K4me3	Histone H3 lysine 4 trimethylation
H3K9/K14ac	Histone H3 lysine 9/14 acetylation
H3S10	Histone H3 serine 10 phosphorylation
H3T22	Histone H3 threonine 22
IBD	Inflammatory bowel disease
IL	Interleukin
IL-1β	Interleukin-1 beta
IL-6	Interleukin-6
IL-18	Interleukin-18
ILCs	Innate lymphoid cells
KDM5	Lysine demethylase 5
lncRNA	Long non-coding RNA
LPS	Lipopolysaccharide
M1/M2	Macrophage polarization states (classically activated/alternatively activated)
mTOR	Mechanistic target of rapamycin
NF-κB	Nuclear factor kappa-light-chain-enhancer of activated B cells
NK	Natural killer
NLRP3	NOD-like receptor family pyrin domain containing 3
PAMPs	Pathogen-associated molecular patterns
PFKFB3	6-Phosphofructo-2-kinase/fructose-2,6-bisphosphatase 3
PRRs	Pattern recognition receptors
Rme2sym	Symmetric dimethylarginine
RNA (RNA-seq)	Ribonucleic acid (RNA sequencing)
RNS	Reactive nitrogen species
ROS	Reactive oxygen species
STAT3	Signal transducer and activator of transcription 3
TAMs	Tumor-associated macrophages
TCA cycle	Tricarboxylic acid cycle
TET	Ten-eleven translocation enzymes
TLRs	Toll-like receptors
TME	Tumor microenvironment
TNF-α	Tumor necrosis factor alpha
UC	Ulcerative colitis
WDR5/MLL1	WD repeat domain 5/Mixed-lineage leukemia 1 complex
